# Pharmacological Inhibition of Feline Immunodeficiency Virus (FIV)

**DOI:** 10.3390/v4050708

**Published:** 2012-04-27

**Authors:** Hakimeh Mohammadi, Dorothee Bienzle

**Affiliations:** Department of Pathobiology, University of Guelph, Guelph, Ontario N1G 2W1, Canada; Email: hmohamma@uoguelph.ca

**Keywords:** antiretroviral therapy, feline immunodeficiency virus, HIV

## Abstract

Feline immunodeficiency virus (FIV) is a member of the retroviridae family of viruses and causes an acquired immunodeficiency syndrome (AIDS) in domestic and non-domestic cats worldwide. Genome organization of FIV and clinical characteristics of the disease caused by the virus are similar to those of human immunodeficiency virus (HIV). Both viruses infect T lymphocytes, monocytes and macrophages, and their replication cycle in infected cells is analogous. Due to marked similarity in genomic organization, virus structure, virus replication and disease pathogenesis of FIV and HIV, infection of cats with FIV is a useful tool to study and develop novel drugs and vaccines for HIV. Anti-retroviral drugs studied extensively in HIV infection have targeted different steps of the virus replication cycle: (1) inhibition of virus entry into susceptible cells at the level of attachment to host cell surface receptors and co-receptors; (2) inhibition of fusion of the virus membrane with the cell membrane; (3) blockade of reverse transcription of viral genomic RNA; (4) interruption of nuclear translocation and viral DNA integration into host genomes; (5) prevention of viral transcript processing and nuclear export; and (6) inhibition of virion assembly and maturation. Despite much success of anti-retroviral therapy slowing disease progression in people, similar therapy has not been thoroughly investigated in cats. In this article we review current pharmacological approaches and novel targets for anti-lentiviral therapy, and critically assess potentially suitable applications against FIV infection in cats.

## 1. Introduction

The feline immunodeficiency virus (FIV) was first isolated in Petaluma, California, from cats with an immunodeficiency-like syndrome [1,2]. FIV is an enveloped virus and, similar to other lentiviruses, has a virion diameter of 105–125 nm and includes a host cell-acquired membrane with viral glycoproteins protruding as spike-like projections [1,3]. As in other complex retroviruses, the FIV genome includes the three large open reading frames (ORF) *gag*, *pol*, and *env*, and several genes that code for small accessory proteins such as Vif and Rev. The three large ORFs code for: (1) the structural polyprotein Gag, comprised of a myristoylated matrix (MA, p15) protein, a capsid (CA, p24) protein and a nucleocapsid (NC, p13) protein; (2) the viral polymerase (POL) protein containing reverse transcriptase (RT), integrase (IN), protease (PRO) and deoxyuridine pyrophosphatase (DU) enzymes; and (3) the Env protein comprised of the heavily glycosylated surface unit (SU) protein gp95 and the transmembrane (TM) protein gp40 [2,4]. 

**Figure 1:**
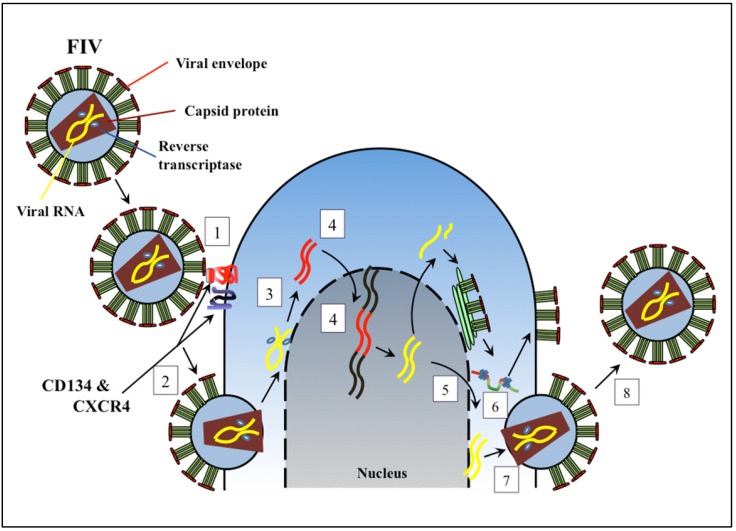
Diagram of Feline immunodeficiency virus (FIV) replication with sites for potential antiretroviral therapy indicated: (1) Viral attachment to cell surface receptors; (2) Viral fusion with the cell membrane; (3) Reverse transcription; (4) Nuclear translocation and integration of into host genome; (5) Viral transcription and nuclear export; (6) Viral protease and protein processing; (7) Virion assembly and maturation; (8) Virion release.

FIV infects lymphocytes, cells of the monocyte/macrophage lineage, and cells of the central nervous system. The viral replication strategy is highly similar to that of HIV, and is initiated by interaction of the viral Env glycoprotein with CD134, a molecule up-regulated on activated CD4^+^ T cells [5]. This interaction exposes previously masked Env epitopes that bind with high affinity to the chemokine receptor CXCR4, which permits viral and cell membrane fusion and subsequent viral nucleocapsid entry into the host cell cytoplasm (Figure 1) [6]. Hence, although FIV Env does not interact with CD4, the primary receptor for HIV, CD4^+^ T cells are nevertheless targeted due to utilization of a receptor with highest expression on memory CD4^+^ T cells. The viral entry receptor, CXCR4, is utilized by both HIV and FIV, and, if present at high density, may facilitate viral entry without initial interaction with CD134 [7]. The latter mechanism might account for infection of CD134 negative cells, such as microglia. Following cell entry, viral RNA released into the cytoplasm, transcribed to complementary DNA, synthesized to viral double-stranded DNA, and transported into the nucleus for integration into the host genome by action of viral IN (Figure 1). FIV Rev assists with transport of viral mRNA, and the Orf A protein may contribute to viral release from infected cells [4,8]. Viral mRNA and genomic RNA are then transcribed, and transported to the cytoplasm for mRNA translation to viral proteins. The immature virion moves to the cell membrane, acquires the viral envelope and glycoproteins, and is then released from infected cells [9,10]. 

Identifying effective antiretroviral therapy (ART) has been of paramount importance since the beginning of the HIV epidemic approximately 30 years ago. The first antiretroviral agent, the RT inhibitor zidovudine, was administered to patients soon after discovery of HIV [11]. Since then, intense effort has been devoted to developing additional drugs with higher efficacy and lower toxicity, suitable for prevention of infection, and applicable in unique patient populations such as pregnant women and those with viral co-infections. In general, access to modern combination ART has turned an invariably fatal infection into a chronic but manageable condition [10,12,13]. However, ART is of limited availability to the majority of HIV-infected persons living in resource-restricted regions of the world. 

At present, approximately 30 compounds are approved by the US Food and Drug Administration (FDA) for treatment of different stages of HIV infection. Types of anti-retroviral drugs include fusion or entry inhibitors, nucleoside reverse transcriptase inhibitors (NRTI), non-nucleoside RT inhibitors (NNRTI), nucleotide RT inhibitors, viral IN blockers and PRO inhibitors (Table 1) [14,15,16,17]. However, rise in resistance among circulating strains of HIV, and side-effects of available drugs, drive the persistent need to discover new therapeutics [10,12,13,14,18]. Table 2 summarizes antiretroviral compounds under development. 

Antiretroviral therapeutic agents have been tested to a limited extent against animal retroviruses. However, FIV genome organization, protein chemistry and the pathogenesis of infection are very similar to HIV [3], rendering the FIV-cat model highly suitable for testing many types of antiviral compounds and strategies. Furthermore, the availability of molecular tools, reagents and *in vitro* and *in vivo* test systems is substantial and increasing, which allows detailed assessment of immune responses, viral parameters and adverse effects in this relatively large and outbred animal model [19,20,21]. Here, we review antiretroviral compounds in use or in development, their mechanism of action, their safety in cats, and their efficacy against FIV. 

**Table 1:** Antiretroviral drugs approved for treatment of human immunodeficiency virus (HIV) infection.

Nature of interference	Agent
Virus entry	Maraviroc (CCR5 antagonist), Enfuvirtide (fusion inhibitor)
Nucleoside reverse transcriptase inhibition	Zidovudine, Stavudine, Lamivudine, Didanosine, Abacavir, Emtricitabine
Nucleotide reverse transcriptase inhibition	Tenofovir disoproxil fumarate
Non-nucleoside reverse transcriptase inhibition	Efavirenz, Nevirapine, Delavirdine, Etravirine
Protease inhibition	Indinavir, Ritonavir, Nelfinavir, Saquinavir, Atazanavir, Darunavir, Fosamprenavir, Tipranavir, Lopinavir
Integrase inhibition	Raltegravir

**Table 2:** Some anti-HIV drugs under development.

Approach	Agent
CD4 attachment inhibition	PRO-542, BMS-806
Chemokine receptor inhibition	Vicriviroc, PRO 140
Fusion inhibition	ADS-J1
Nucleoside reverse transcriptase inhibition	Emtricitabine, Amdoxovir
Non-nucleoside reverse transcriptase inhibition	DPC-083, Etravirine, Calanolide A
Protease inhibition	Darunavir
Integrase inhibition	Elvitegravir, MK-2048

## 2. Inhibitors of Virus Entry

### 2.1. Attachment Inhibitors

Inhibitors of viral attachment bind to receptors on the host cell membrane to obscure the site of interaction of Env with receptor (Figure 1, step 1) [10,14,22]. Since positively charged regions in the V3 loop of HIV gp120 non-specifically attach to negatively charged heparan sulphate proteoglycan (HSPG) and lectin receptors on host cells, polyanionic or oligosaccharide compounds such as heparin, dextran sulphate, and high-mannose carbohydrates can block *in vitro* virus adhesion [10,14]. Polyanionic compounds were also reported to *in vitro* reduce FIV replication and syncytium formation [23], and the sites in the FIV Env interacting with HSPG have been mapped [24]. The latter data showed that tissue-culture adapted FIV strains such as 34TF10 differed from FIV field strains such as PPR by mutations in the V3 loop of the envelope. These mutations change glutamine to lysine, which favours binding to HSPG [24]. Additional sites in the V3 loop also contributed to the interaction, and envelopes binding to HSPG had lesser dependence on prior interaction with CD134 for cell entry via CXCR4. Non-specific attachment mechanisms may modulate cell tropism and viral entry under specific *in vivo* circumstances, but data showing *in vivo* efficacy of HPSG or lectins to reduce virus infection or load are sparse. Nevertheless, considering that FIV and HIV envelopes are abundantly glycosylated, and that 2G12 (a broadly HIV-neutralizing antibody) recognizes a carbohydrate epitope, suggests that binding of viral glycoproteins to lectin receptors is an aspect of the host-pathogen interface to be further explored for therapeutic and preventive approaches [25,26]. 

The type of host cell employed for lentivirus propagation affects inhibition by plant-derived lectins. For example, the carbohydrate binding compounds of *Galanthus nivalis* (snowdrop) agglutinin (GNA) and *Hippeastrum hybrid* (amaryllis) agglutinin (HHA) prevented HIV entry by binding to mannose residues on the viral Env glycoproteins [27]. Both GNA and HHA were tested for inhibition of FIV derived from Crandell-Reese feline kidney (CRFK) cells and dendritic cells (DCs) co-cultured with T cells. FIV grown in CRFK cells was more sensitive to inhibition by both GNA and HHA compared to virus grown in blood mononuclear cells and DC-T cell co-cultures. Differential sensitivity to lectins likely resides in variable composition of oligosaccharides of the FIV envelope glycoproteins, which in turn may reflect differences in post-translational processing between epithelial cells and leukocytes [28].

Agents that specifically block interaction of gp120 with CD4 are soluble CD4 receptors and antibodies directed against CD4. The former proved too short-lived in circulation to be practical, but ibalizumab, a non-immunosuppressive monoclonal antibody to CD4, is in phase II clinical trials for treatment in patients with HIV resistant to conventional therapy [29]. Initial results showed reduced patient viral loads and limited development of resistance due to changes in viral envelope glycosylation [14,29]. Blocking the initial interaction of gp120 with CD4 may not only reduce HIV cell entry and decrease apoptosis, but also drive viral selection toward CXCR4 chemokine receptor usage [30]. Analogous compounds blocking the interaction of FIV gp95 with CD134 have not been reported, although sites of gp95 binding to CD134 have been mapped in detail [7,31]. Similar to the interaction of HIV gp120 with CD4, the site where FIV gp95 binds CD134 is distinct from the ligand binding site, and soluble CD134 constructs may induce conformational changes in gp95 to expose a cryptic CXCR4 site [6,7].

### 2.2. Inhibitors of Co-receptor Interaction

Proteins, small molecules and antibodies have been investigated for inhibition of viral entry via the chemokine receptors CCR5 (HIV) and CXCR4 (HIV and FIV) [10,14,32]. Both chemokine receptors are transmembrane G-protein coupled receptors whose main endogenous ligands are RANTES and stromal-derived factor-1 (SDF-1), respectively. CCR5 antagonists were in clinical development for other indications when it was identified that naturally occurring lack of CCR5, due to a homozygous deletion mutant, imparted marked resistance to HIV infection but did not adversely affect immune function [33]. However, attempts to subsequently devise potent and specific CCR5 antagonists with favourable pharmacokinetic properties proved challenging. TAK779 was an early non-peptide CCR5 antagonist, but had limited oral bioavailability [34]. Three other CCR5 antagonists, aplaviroc, vicriviroc and maraviroc, were tested in clinical trials in people. Maraviroc had favourable efficacy and pharmacokinetics, and was approved for use in treatment-experienced HIV patients [17]. Additional CCR5 antagonists are under development. Concern over potential emergence of CXCR4-tropic HIV strains in people treated with CCR5 antagonists spurned efforts to also develop antagonists to CXCR4. However, since interaction of SDF-1 with CXCR4 is a key regulator of hematopoietic cell homeostasis and vasculogenesis, and blockade of this interaction is employed in cancer therapy, very specific interference with HIV envelope binding was required [35]. Plerixafor (AMD-3100), a synthetic macrocyclic compound, and other CXCR4 antagonists, effectively block the interaction of gp120 with CXCR4. Limited oral bioavailability and short half-life preclude clinical use of plerixafor in HIV infection, but additional CXCR4 antagonists are under development [36]. 

The amino acid sequence of human and feline CXCR4 is highly similar, and either species’ molecule can facilitate fusion of HIV and FIV with cells [37], hence CXCR4 antagonists developed against HIV may also be of use against FIV. AMD-3100 effectively blocked *in vitro* FIV gp95 binding to CXCR4 [38], and anecdotal reports suggest the compound was well tolerated in cats [39], hence further assessment of the clinical utility of CXCR4 antagonists in cats appears warranted.

### 2.3. Fusion Inhibitors

Fusion inhibitors prevent entry of retroviruses. These inhibitors are either synthetic peptides that mimic a part of gp41 or small molecules that bind to gp41, and both eventually block interaction of uncovered envelope glycoprotein gp41 with the cell membrane (Figure 1, step 2). Fusion inhibitors have certain advantages over other antiretroviral agents because of their extracellular site of action and effect on viral but not cellular proteins. Therefore, they are generally of lower drug-associated toxicity [13,40]. The first FDA-approved drug in this category was enfuvirtide (T-20), a synthetic peptide of 36 amino acids that mimics a 7-repeat hydrophobic region near the N-terminus of gp41. Binding of enfuvirtide to gp41 prevents formation of a trimeric hairpin necessary for fusion and entry of HIV into the host cell [41]. A synthetic peptide to an analogous hydrophobic region of FIV gp40 prevented viral entry into CRFK cells, and into HeLa cells expressing feline CXCR4, and reduced viral replication in lymphoid cells [42]. Similarly, other peptides to comparable regions in FIV gp40 also reduced viral fusion [43,44]. A synthetic retroinverso octapeptide of the FIV transmembrane glycoprotein containing the Trp-rich motif has high affinity for FIV gp40 and has been demonstrated to have inhibitory effects on FIV replication *in vitro*. It was well tolerated by cats and markedly reduced FIV replication in chronically FIV-infected cats [45,46]. Additional HIV gp41 fusion inhibitors are under development [47], but a relative lack of stability, need for delivery by subcutaneous injection, short *in vivo* half life, and acquired resistance due to mutations, have limited their use to specific scenarios of treatment failure with other antiretroviral drugs. Similar concerns would likely also limit use in cats, but *in vivo* evaluation of FIV peptide fusion inhibitors, that are highly effective *in vitro*, remains to be undertaken. 

## 3. Inhibitors of Reverse Transcription of Viral Genomic RNA

Reverse transcriptase inhibitors (RTI) are divided into three categories: nucleoside, nucleotide, and non-nucleoside reverse transcriptase inhibitors (NRTIs, NtRTIs and NNRTIs, respectively). Each type of compound blocks the catalytic activity of viral RT by a slightly different mechanism. Because of its essential role in the viral life cycle, RT has been a major target of ART since the discovery of HIV (Figure 1, step 3).

### 3.1. NRTIs and NtRTIs

NRTIs are analogues of natural deoxynucleotides (dNT) but lack a 3’- hydroxyl group. NRTIs are metabolized to their active tri-phosphorylated form by cellular kinases and then compete with cellular dNTs for incorporation into growing strands of proviral DNA being synthesized by viral RT. However, since NRTIs lack a 3’-hydroxyl group, the next dNT cannot form a phosphodiester bond and DNA elongation is terminated (Figure 1, step 3). Zidovudine (azidothymidine, AZT) was the first NRTI agent approved for the treatment of HIV infection, and since then at least 7 additional NRTIs were approved for treatment of HIV, and, in some cases, hepatitis B infection.

NtRTIs are structurally similar to NRTIs but contain a phosphonate group and do not require further phosphorylation by cellular enzymes. Therefore, NtRTIs are already in active form for incorporation into growing strands of viral DNA, and their mechanism of action is similar to that of NRTIs. Tenofovir disoproxil fumarate (TDF) is the only NtRTI approved for treatment in HIV/AIDS patients [14]. 

NRTIs have been assessed for *in vitro* and *in vivo* inhibition of FIV replication. Compounds investigated were zidovudine and acyclic nucleoside phosphonates such as 9-(2-phosphonylmethoxyethyl) adenine (PMEA) and (*R*)-9-(2-phosphonylmethoxypropyl) adenine (PMPA). Zidovudine prevented up to 95% of FIV replication in CRFK cells [48]. In another study, PMEA had better inhibitory efficacy than 2', 3’-dideoxyadenosine (ddA) on FIV_Pet_ replication in CRFK cells [49,50]. However, of note, cell systems for assessment of anti-FIV activity may markedly affect outcomes: the antiviral activity of three NRTIs (PMEA, 9-(2- phosphonylmethoxypropyl) diaminopurine (PMPDAP), and AZT) was rather different in thymocytes relative to DC-thymocyte co-cultures and CRFK cells. This may be because DCs are known to enhance FIV infection, and only viruses independent of interaction with CD134 will infect CRFK cells. Hence, the cell type from which FIV is derived may influence the degree of glycosylation and subsequent infectivity, as well as the production of cell-free virus versus transmission of virus through direct contact of DC with lymphocytes, regardless of susceptibility to RTI. 

PMEA has been administered to a small number of experimentally and naturally infected cats, and reduced FIV replication *in vitro* and *in vivo* up to 200fold [51]. Opportunistic infections were much reduced, and adverse effects from PMEA treatment were not reported [51]. Zidovudine has also been used in FIV-infected cats, and was relatively well tolerated [52]. However, anemia developed in zidovudine-treated cats in a dose-dependent fashion, limiting use of higher dosages. Both compounds resulted in improved CD4/CD8 lymphocyte ratios [53]. FIV replication and viral DNA load in infected cats treated with PMEA for one year were significantly reduced relative to no treatment, but prophylactic administration did not prevent FIV infection in challenged cats [54]. Zidovudine therapy also reduced plasma virus titers in infected cats but did not reduce cell-associated FIV [55]. 

Macrophages are considered to be a reservoir for FIV and HIV that is not readily targeted by drugs due to the relative longevity, limited mitotic activity and unique metabolic profile of macrophages. Hence, macrophages have limited ability to phosphorylate nucleosides such as in NRTIs. Administration of an NRTI, zalcitabine (dideoxycytidine), targeted to macrophages through phagocytosis via *ex vivo* drug-loaded erythrocytes, resulted in reduced macrophage FIV load and relative resistance to infection [56]. However, zalcitabine requires thrice daily administration and has multiple adverse effects in people, and is therefore rarely used. These characteristics suggest zalcitabine should be used cautiously or avoided in cats. 

Fozivudine (FZD), a NRTI intracellularly metabolized to zidovudine, when administered at a dose of 45 mg/kg/12 hours to cats, was not hematotoxic and significantly reduced viral load during acute FIV infection [57]. However, the effect did not persist beyond 4 weeks, which was thought to be due to the development of resistance. Stampidine (d4T), a pyrimidine nucleoside analogue and an aryl phosphate derivative of stavudine, lowered viral RT activity in chronically FIV-infected cats [58]. WHI-07, a derivative of zidovudine, prevented vaginal and rectal transmission of FIV in domestic cats [59]. 

There are several reports of attempted combination antiretroviral therapy for FIV. For instance, Bisset and colleagues showed that abacavir (ABC or 1592U89) not only blocked FIV replication in cell culture, but also acted synergistically with analogues of zidovudine and lamivudine (3TC) to inhibit FIV replication *in vitro* [60]. Arai and colleagues showed that AZT and 3TC together had better efficacy for suppression of FIV replication *in vitro* than either drug by itself [61]. Furthermore, they demonstrated that prophylactic AZT/3TC therapy reduced and delayed infection of cats challenged with FIV. However, AZT/3TC was ineffective in chronically infected cats and high doses were associated with anemia and neutropenia [61].

### 3.2. Non-nucleoside Reverse Transcriptase Inhibitors (NNRTIs)

Non-nucleoside RT inhibitors (NNRTIs) are small molecules that are not natural nucleoside analogues and structurally differ from NRTIs and NtRTIs. Their binding site on the RT enzyme is different than that of NRTIs. NNRTIs attach to a RT domain close to the active site of the enzyme and change the conformation, which results in non-competitive inhibition of catalytic action. There are three HIV NNRTIs that are FDA approved (Table 1); each efficiently inhibits only HIV. Other retroviruses, such as simian immunodeficiency virus (SIV) and FIV, were not susceptible to this type of RT inhibition, presumably because of structural differences in the enzyme binding site [62,63].

## 4. Inhibitors of Nuclear Translocation and Integration of Viral DNA into Host Genome

The hallmark of the retroviral replication cycle is integration of viral double-stranded DNA (ds-DNA) into the host genome. For this process, the pre-integration complex (PIC) is assembled in the cytoplasm of infected cells. The PIC is comprised of two strands of viral RNA, viral IN, and accessory proteins required for nuclear translocation such as viral protein R (Vpr), MA and nucleocapsid (NC) protein, and host proteins [64]. Synthesis of viral dsDNA by RT takes place in the PIC, which is then translocated to the nucleus. Both nuclear translocation and proviral DNA integration have been targets for novel antiretroviral therapy (Figure 1, step 4). Studies targeted to these processes have focused on HIV, but may also be applicable to other retroviruses, including FIV [65]. Protein trafficking from the cytoplasm to the nucleus of eukaryotic cells occurs in either passive or active mode. In passive mode, proteins less than 40 kDa are thought to freely diffuse through nuclear pores [66]. However, translocation of larger proteins from the cytoplasm to the nucleus takes place via an active process which utilizes cellular importins. Importins are proteins that recognize a stretch of basic amino acids, called the nuclear localization signal (NLS), on the cargo protein, and then shuttle such proteins into nucleus [67,68]. HIV proteins such as IN, MA and Vpr contain NLS motifs and contribute to the nuclear import of the PIC [64,69], while inhibitors of nuclear transport aim to interfere with this process. For example, the arylene-bis compound CNI-H0294 binds to the lysine residues in the NLS of MA. By occupying the NLS, CNI-H0294 blocks the interaction of cellular importins with PIC, and prevents nuclear translocation [70]. More recently identified compounds mimic the NLS of MA and by binding to importin α, prevent its interaction with MA [65]. Another novel anti-HIV compound blocked interaction of Vpr with importin α, which prevented HIV replication in primary macrophages [71]. 

Retroviral IN catalyzes the integration of retroviral dsDNA into the host cell genome and interacts with PIC, making it an ideal target for two-site interference by antiretroviral compounds [65,72]. Integrase inhibitors are designed to either foil enzyme activity and therefore viral dsDNA integration, or to prevent formation of the PIC by interfering with the interaction of IN with cellular factors [65]. Transcriptional co-activator p75 (or lens epithelium-derived growth factor, LEDGF) is a cellular protein that not only plays a crucial role as a functional component of the PIC, but also facilitates IN interaction with nuclear chromatin at integration sites after nuclear transport [73]. D77 is a benzoic acid derivative that interrupted IN/LEDGF interaction and HIV IN function, which subsequently prevented viral replication [74]. The IN strand transfer inhibitor naphthyridine carboxamide (L-870810) prevented FIV DNA integration into the host cell genome and resulted in lower virus replication in the feline lymphoid cell line MBM [75,76]. 

## 5. Inhibitors of Viral Transcription and Nuclear Export

Generation of progeny viral mRNA and transfer to the cytoplasm of the host cell are the next steps in the retroviral replication cycle (Figure 1, step 5). Transcription of proviral DNA is controlled by the flanking long terminal repeats (LTR). The 5’ LTR initiates transcription and the 3’ LTR is essential for polyadenylation of the transcripts. Lentiviral accessory proteins such as HIV Tat, Rev and Vpr, and FIV Vif and Vpr, as well as cellular factors such as NF-ĸB, control viral transcription. Therefore, the accessory proteins have also been of interest as targets of antiviral therapy [14]. The transfer of HIV mRNA to the cytoplasm is dependent on the regulatory protein Rev, which binds to a Rev responsive element (RRE) in the viral mRNA. Rev contains both NLS and nuclear export signal (NES) motifs that enable shuttling of the protein between cytoplasm and nucleus. NES is a leucine-rich sequence required for the nuclear export of proteins via the CRM1/exportin 1 protein [14]. The interaction of Rev with RRE can be disrupted with tetracationic diphenylfuran, which binds to specific nucleotides in the RRE and blocks binding of Rev [77]. There are also compounds that directly bind to the NES binding site in CRM1 and prevent interaction with Rev. For example, compound PKF050-638 binds to Cys-539 of CRM1 and prevents CRM1-mediated Rev nuclear export [78]. The course of action of PKF050-638 and other inhibitors of CRM1 is similar to leptomycin B, which was the first agent identified as preventing nuclear export of Rev by blocking CRM1 [79]. Leptomycin, therefore, had anti-HIV activity, but toxicity in cell culture precluded clinical application [79]. Other CRM1 blockers of Rev nuclear export, such as 5,6-dihydrovaltrate, may also be effective in reducing HIV assembly in the cytoplasm, and are promising new agents for anti-retroviral therapy [80].

## 6. Inhibitors of Viral Protease and Virion Assembly

The retroviral protease (PRO) has a pivotal role in processing Gag and Gag-Pro-Pol polyproteins to functional proteins necessary for maturation of virus particles [81]. Since PRO plays a key role in the viral replication cycle, it has been of great interest to develop inhibitors for this enzyme (Figure 1, step 6). The retroviral PRO is a homodimer with the active site located at the dimer interface. Protease inhibitors (PI), together with RT inhibitors, are two key components of combination anti-retroviral therapy in HIV patients. The initial PIs were designed to competitively bind to the active site of the enzyme, and therefore to inhibit access to viral protein substrates. The ten PIs that were first FDA approved all were competitive inhibitors (Table 1) [82]. Since the emergence of viral strains resistant to PIs, new approaches focused on designing compounds to bind at sites other than the active site of the enzyme. Through a fragment-based screening approach, several compounds were selected for their strong binding affinity to different sites of the enzyme and destabilization of enzyme conformation [83]. A specific compound, screened out of a library of inhibitors of protein-protein interactions, was able to inhibit the activity of wild-type PRO and that of six PI-resistant mutants [83,84]. 

In common with other retroviruses, the FIV PRO also cleaves viral Gag and Gag-Pol, yielding nine structural and non-structural proteins of FIV including MA, CA, NC, PRO, RT and IN [85]. There are a few studies on the effect of PIs on FIV replication and disease control. TL-3, a PI that binds to the active site of the FIV PRO, completely prevented virus production in cell culture [86]. In an *in vivo* study, TL-3 prevented FIV-induced neurological degeneration when administered at the early stages of infection in cats, but did not prevent FIV viremia in challenged cats [87]. In an *in vitro* study, the effects of three HIV PIs on FIV_Pet_ replication in MBM cells were investigated. Results indicated that all three PIs (tipranavir, atazanavir, and lopinavir) inhibited FIV replication, but only tipranavir was comparably effective against FIV and HIV [88]. 

The maturation of virions occurs as the result of conformational changes in CA protein molecules that have been assembled (Figure 1, steps 7 and 8). Compounds and peptides that target the C-terminal or N- terminal domains of the CA can block the required interaction for assembly of virions. Lack of CA assembly, in turn, inhibits viral replication by blocking the final cleavage of Gag protein, which precludes virus maturation. Beviramat has preventative effects on HIV replication *in vitro* via this mechanism. Beviramat is the only HIV assembly inhibitor that has been used in clinical trials, but has yielded poor responses [89,90]. 

## 7. Miscellaneous

Several host cell proteins and innate immune cytokines have antiretroviral effects. TRIM5 (a member of the tri-partite motif family of proteins) is a host protein that in some species binds to the retroviral CA in the cytoplasm, generating CA-TRIM5 complexes, which prevents retrovirus uncoating and enhanced proteasomic degredation. Cyclophilin A (a member of the peptidyl proline isomerase superfamily) contributes to retroviral restriction by interacting with TRIM5, and a fusion protein of feline TRIM5 and cyclophilin A *in vitro* prevented FIV and HIV infection [91,92]. The TRIM5/cyclophilin fusion protein bound the FIV capsid, and enhanced targeting to the proteasome, thereby preventing reverse transcription [93]. Therapy with such constructs may comprise a genetic approach to treatment of lentiviral infection. Additionally naturally occurring restriction factors and antiviral proteins have been described in cats and other species, but have not yet been employed for antiviral therapy.

Interferon omega had inhibitory effects against FIV *in vitro*, and is licensed to use in FIV-infected cats in some European countries and in Japan. Interferon alpha has also been suggested to improve health and survival of FIV positive cats [52]. 

## 8. Conclusions

Current antiretroviral agents are mostly studied for their effects on HIV. However, because of persistent emergence of drug resistant HIV strains, toxicity of many agents, and the requirement for lifetime therapy, the search for new less toxic and highly efficacious agents continues. Animal models may be very helpful for assessing antiviral properties, and among lentiviruses, FIV infection of cats is a well-established model. However, structural differences in viral proteins, and distinct differences between primates and cats in xenobiotic disposition, restrict the utility of the model to certain agents (Table 3).

**Table 3:** Antiretroviral drugs evaluated for inhibition of FIV replication.

Approach	Compounds	Outcome
CXCR4 entry inhibitor	AMD3100	Reduced FIV replication *in vitro* and tolerated by cats [38,39].
Nucleoside reverse transcriptase inhibitor	Zidovudine, Stavudine, PMEA, Dideoxycytidine, Fozivudine, WHI-07 (derivative of zidovudine), Stampidine, Lamivudine	Prevented virus replication *in vitro* and *in vivo*, decreased FIV load in chronically infected cats, tolerated [48,49,50,51,52,53,54,55,56,57,58,59,60,61].
Protease inhibitor	Atazanavir, Tipranavir, Lopinavir, TL-3	Inhibited FIV replication, reduced neurodegeneration and *in vivo* tolerated [86,87,88].
Integrase inhibitor	L-870810	Reduced FIV replication *in vitro* [75].
